# 2213. Increased Early Syphilis Detection and Treatment in an Urban Emergency Department During the COVID-19 Pandemic

**DOI:** 10.1093/ofid/ofac492.1832

**Published:** 2022-12-15

**Authors:** Ashley Lipps, Carlos Malvestutto, Susan L Koletar, Michael Dick, Sommer E Lindsey, Jose A Bazan

**Affiliations:** The Ohio State University Wexner Medical Center, Columbus, Ohio; The Ohio State University Wexner Medical Center, Columbus, Ohio; Ohio State University, Columbus, Ohio; THE OHIO STATE UNIVERSITY, COLUMBUS, Ohio; Ohio State University, Columbus, Ohio; The Ohio State University, Columbus, Ohio

## Abstract

**Background:**

Many people at risk for sexually transmitted infections (STIs) utilize the emergency department (ED) for evaluation and treatment of STI-related complaints. Syphilis testing rates in the ED have historically been low. Following the implementation of a joint ED and Infectious Disease (ID) multidisciplinary quality improvement initiative designed to increase syphilis testing in this setting, we sought to evaluate the demographics and clinical outcomes for patients diagnosed with early syphilis in the ED.

**Methods:**

A retrospective chart review of patients with positive syphilis test results from ED encounters at the Ohio State University Wexner Medical Center East Hospital between 1 January 2019 and 31 December 2021 was performed. Demographic and clinical information was obtained for patients diagnosed with early syphilis (primary, secondary or early latent syphilis).

**Results:**

Since 2019, we have observed a significant increase early syphilis cases identified in our ED despite stable rates of testing (Figure 1). There were 55 cases of early syphilis, 3 with neurosyphilis (NS) at the time of diagnosis (Figure 2). Most cases were men (38/55; 69%), Black/African American (44/55; 80%), and symptomatic (48/55; 87%). Nine of 55 patients (16%) had HIV co-infection, 2 were new diagnoses at time of syphilis diagnosis. Most patients (44/55; 80%) completed appropriate treatment. Of the symptomatic patients, 17/45 (37%) received presumptive treatment in the ED (3 cases of NS excluded). Of those who received treatment after discharge from the ED, the median time to treatment completion was 3 days (range 0-30), with 11/24 (46%) returning to the ED for treatment.

**Figure 1.**
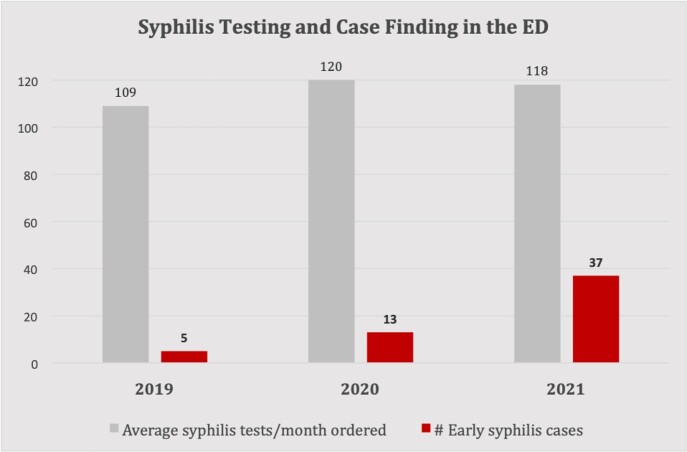

**Figure 2.**
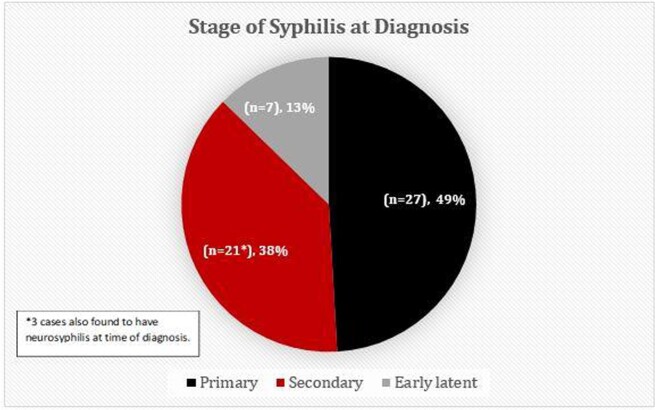

**Conclusion:**

As a result of a collaborative ED/ID STI testing initiative established at our institution, we have observed a significant increase in the number of symptomatic early syphilis cases identified in our ED between 2019 and 2021, with most patients completing treatment in a timely manner. This data show that the ED is an important and feasible setting for syphilis diagnosis and treatment when appropriate support systems are in place, including established communications with ID colleagues.

**Disclosures:**

**Carlos Malvestutto, MD MPH**, Gilead Sciences: Advisor/Consultant|Viiv Healthcare: Advisor/Consultant **Susan L. Koletar, MD**, Gilead Sciences: Grant/Research Support.

